# Phasic alertness boosts representational momentum

**DOI:** 10.3389/fpsyg.2022.1003661

**Published:** 2022-11-29

**Authors:** Junjie Yan, Jingwen Zeng, Peiduo Liu

**Affiliations:** Faculty of Psychology, Southwest University, Chongqing, China

**Keywords:** representational momentum, phasic alertness, executive control, warning cue, retention interval

## Abstract

The final location of a moving object is always misremembered in the direction of the object’s motion; this occurrence is called representational momentum. Three experiments were conducted to investigate the effects of phasic alertness on representational momentum by presenting a visual or auditory warning cue. In experiment 1, the mouse pointer paradigm was used, and the results showed that external warning cues increased forward displacement. Experiment 2 indicated that the effects of phasic alertness and speed of motion on representational momentum were independent. In experiment 3, the probe paradigm was used, and the results showed that external warning cues increased forward displacement as well as participants’ sensitivity to the difference between the target and probe positions. These findings prove that phasic alertness boosts rather than reduces representational momentum. We propose that phasic alertness might influence representational momentum by modulating the process of executive control in the retention interval.

## Introduction

Even extremely simple actions in daily life are subject to varied motions and localizations. However, humans do not localize moving objects with complete accuracy. When a moving target vanishes suddenly, its final location is always displaced along the direction of its motion, which is called representational momentum (Freyd and Finke, 1984). Typical studies of this phenomenon show observers three rectangles whose rotation angles increase in turn to induce an implicit motion representation and present a probe stimulus near the third rectangle. Observers are asked to determine whether the location of the probe is the same as where the third rectangle vanished (Teixeira et al., 2019; Merz et al., 2020, 2021). The results of these studies show that the assessment responses are more prone to be the “same” when the angle of the probe is actually larger, which suggests that the memory of the location where the third rectangle vanished is displaced along the direction of rotation. In addition to implicit motion, representational momentum occurs in actual smooth motion (Hubbard and Bharucha, 1988); thus, representational momentum is a common localization bias. Researchers have found that this displacement is influenced by a number of variables (for reviews, see Hubbard, 2005, 2014, 2017) which in general are divided into four categories: (a) target, (b) display, (c) context, and (d) observer. The last category—observer—has rarely been discussed, particularly how the characteristics of observers affect visual localization. Among these characteristics, attention is a factor in the visual localization process that warrants further exploration.

Attention plays a crucial and irreplaceable role in a variety of human cognitive behaviors, such as stimulus extraction and encoding, information selection and filtering, memory, speech performance, and thinking. In general, attention can be divided into three components: alertness, orienting, and executive control (Peterson and Posner, 2012). Alertness is readiness for upcoming events with a high arousal level. Orienting, which defines the ability to select and filter information among multiple stimuli, is the most studied component. Executive control refers to the ability of people to monitor conflicts and plan, select, inhibit, and execute responses (Posner and Peterson, 1990; Peterson and Posner, 2012; Dash et al., 2019). Due to the diversity of attention components and the different functions of each component in cognitive behaviors, when we discuss the effect of attention on representational momentum, it is always necessary to distinguish the component that is the focus of the discussion.

In the past 2 decades, some researchers have investigated how attention influences representational momentum. Hayes and Freyd (2002) designed two dual-task experiments to distract observers’ attention in representational momentum tasks. In experiment 1, they presented a fixation at the central point, a moving dot above the fixation and a varied square that could grow or shrink in size below the fixation. After three frames of presentation, they showed observers one probe, which could detect either the dot’s final position or the square’s final size. In some blocks, there were more trials in which the dot’s final position was probed than trials in which the square’s final size was probed, e.g., proportions of 80 and 20%, respectively, while in other blocks, the opposite distribution was used, e.g., 20–80%. The researchers presumed that the object that was more frequently probed would receive more attention. The results showed that when the dot was less attended, the forward displacement (FD) increased, whereas when the square was less attended, observers were more prone to remember the square as smaller than it actually was. The researchers considered that the dot results indicated that attention inhibits representational momentum, while the square results were consistent with the kind of memory bias reported by Hubbard (1996). This bias suggested that individuals would remember the square’s final size as smaller when it is growing or shrinking. They thought that this bias would become stronger when observers’ attention was distracted. In experiment 2, only the dot was presented, and the observers were asked to complete a counting task. They found a larger FD of the dot when the observers’ attention was distracted by the counting task. The researchers concluded that attention is required to halt FD, which suggests that representational momentum is an automatic process. In contrast, Kerzel (2003a) presented distractors on the top, bottom, left, and right of the final target position in the retention interval and found that the displacement decreased or even reversed when distractors were presented. Therefore, Kerzel suggested that attention boosts representational momentum and is required to maintain mental extrapolation.

It seems that the conclusions of Hayes and Freyd (2002) and Kerzel (2003a) regarding the relationship of attention and representational momentum contradict each other. However, given that the methods these researchers used to manipulate attention were different—Hayes and Freyd manipulated attention during presentation of the target, but Kerzel did so in the retention interval—the discrepancy may have been caused by the methods rather than by attention *per se*. This difference was discussed in detail in a later study (Hubbard et al., 2009). Hubbard et al. presented a spatial cue during the target motion or retention interval that was located in the true position where the target would vanish or above the true position. The results showed that regardless of whether the cue was presented during the target motion or retention interval, cue presentation decreased FD. The authors suggested that the cue presented during target motion functioned as a prime to allow readiness of the observers in advance. In this case, the allocation of attention to the final position of the target increased and further decreased FD, consistent with the findings of Hayes and Freyd (2002). In contrast, the cue presented during the retention interval functioned as a distractor whose effect was consistent with findings of Kerzel (2003a). This distractor might not influence attention *per se*, but instead disrupt or even eliminate the representation of the final location of the target; thus, the distractor would not reflect the effect of attention on representational momentum. Therefore, Hubbard et al. agreed that attention inhibits representational momentum and is required to halt mental extrapolation.

The study by Hubbard et al. (2009) may suggest a possible interpretation that could resolve the contradiction on the effect of attention on representational momentum, but two unresolved problems remain. First, Hubbard et al. regarded the cue presented during target motion as a prime that caused observers to be ready in advance, so the cue might not only increase individuals’ attention to the final location of the target but could also lead to an alertness to the target’s vanishing. In fact, due to the fixed interval between each pair of stimuli, when the cue was presented, observers might tend to infer how many stimuli would appear until the probe showed up according to the distance between the target and the cue; this created a state similar to a countdown. Even though this countdown effect could occur without the cue, the cue obviously enhanced it; moreover, the position where the probe would appear was uncertain, so this task involved the same characteristics as those of alertness: a state of readiness or vigilance for an upcoming stimulus, event, or reaction (Posner and Peterson, 1990; Peterson and Posner, 2012; Li et al., 2017). However, any advance indication about the timing or location of stimuli should not be involved in alertness (Weinbach and Henik, 2012); nevertheless, the cue in the study of Hubbard et al. did hint at the final location of the target. This could allow observers to improve the accuracy of their responses by strengthening the memory of the final location of the target, which could also decrease FD. Therefore, the decreases in FD were caused by alertness or location hints as well; and it is still unclear how representational momentum is influenced by alertness. Second, for the cue presented during the retention interval, which is consistent with the distractors used in study of Kerzel (2003a), Hubbard et al. suggested that the cue reflected a significant disruption or even elimination of the representation of the final location of the target rather than attention *per se*; nevertheless, it is still unclear why FD would decrease rather than increase after disruption or elimination caused by distractors. In general, alertness may play a role in these two unresolved problems. Therefore, the present study investigated how alertness affects representational momentum in several experiments.

Alertness, as one of the three components of attention, is often considered the basis of orienting and executive control (Peterson and Posner, 2012; Wiegand et al., 2017; Haupt et al., 2019; Poth, 2020). Two types of alert system have been described. Tonic alertness is a top-bottom continuous preparation ability in the absence of external cues, whereas phasic alertness refers to a bottom-top ability that is used to create a state of vigilance and readiness for an upcoming event after an external cue has been presented (Peterson and Posner, 2012; Haupt et al., 2018; Nakagawa and Sukigara, 2019). Past studies have shown that alertness could lower observers’ threshold of conscious perception (Botta et al., 2014; Chica et al., 2016; Olah et al., 2020) and facilitate visual processing in general (Petersen et al., 2017; Wiegand et al., 2017). Moreover, various interactions between alertness and executive function have been found. Executive function has been proved to be associated with vigilance decrement (Luna et al., 2018, 2022b), and phasic alertness can enhance the global and automatic processing of visual stimuli and further inhibit executive control performance (Weinbach and Henik, 2011; Weinbach et al., 2015; Tanzer et al., 2016; Zani and Proverbio, 2017).

The present study mainly focused on phasic alertness, which was prompted by an external cue presented during target motion. Both visual and auditory cues were used in the experiments, given that the alertness states caused by cues from different channels may differ. The visual cue warrants special attention because it must not contain any time or location information; thus, the cue was designed to appear inside the target and move synchronously with the target. In addition, a smooth motion paradigm was used because the certainty of interval time and the distance of stimuli in the implicit motion paradigm make it difficult to set any warning cue straightforwardly. Past studies have shown that FD is smaller in smooth motion than in implicit motion (Kerzel, 2003b), but this detail did not hinder the discussion of the effect of alertness on FD. In experiment 1, the mouse pointer paradigm was used to investigate whether phasic alertness can influence representational momentum. In experiment 2, the modulation of the influence of phasic alertness on representational momentum by the speed of motion of the target was explored. In experiment 3, the probe paradigm was used to further confirm the result and discover the effects of phasic alertness on other measurements in addition to FD.

## Experiment 1

The question of experiment 1 was whether simple phasic alertness can influence representational momentum. The mouse pointer paradigm was used in experiment 1. Phasic alertness was produced by a visual or auditory cue presented in the stage of target movement. If phasic alertness reduces representational momentum, then FD should be smaller in the trials with visual or auditory cues present than in trials with cues absent in the same block. If phasic alertness boosts representational momentum, then we should obtain the opposite results for FD.

### Methods

#### Participants

Twenty-eight right-handed students (21 females, mean age 21 ± 1.41 years) from Southwest University of China were paid for their participation. All had normal or corrected-to-normal vision. None reported any visual or auditory impairment. Before commencing the experiment, they signed a consent protocol approved by the Ethics Committee of Southwest University. The result of the *post-hoc* power analysis showed 1−β>0.99 for the observed main effect of the cue with FD as dependent variable [ f=0.83,α=0.05,N=28 , run with G*Power 3 (Luna et al., 2022b)].

#### Stimuli and apparatus

In experiment 1, the target stimulus was a white ball (1.15° × 1.15°) that moved along the horizontal centerline against a black background. The visual cue was a triangle warning sign that would appear in the center of the target ball, whereas the auditory cue was a pure tone (1,600 Hz, 50 dB) delivered *via* EDIFIER K815 headphones. The experimental program was written with the TKINTER toolbox in Python 3.7 and appeared on a 19-inch LED monitor (DELL P1917S) whose resolution was 1,280 × 1,024 and refresh rate was 60 Hz.

#### Design and procedure

The observers viewed a white ball moving leftward or rightward and used a mouse to click the position where the ball vanished (Kerzel, 2004). The warning cues appeared and later vanished during target motion. The observers completed two blocks: a visual block in which visual cues were present in half of the trials and an auditory block in which auditory cues were present in half of the trials. Taking the visual block as an example, the independent variable was the warning cue (absent vs. present), and each block had 20 repetitions. The target ball moved rightward in half of the 20 trials and leftward in the other half of the trials. The dependent variables were FD and response time (RT). To prevent premature responses, there was an extra 5% of catch trials in which the mouse pointer did not appear after the target ball vanished, thus skipping the response stage (Jongen and Smulders, 2007; Levin et al., 2011; Kennefick et al., 2014). Overall, each block had 42 trials, and the experiment had 84 trials in total. The observers rested for 2 min between the two blocks, and the order of the two blocks was balanced among the observers.

**Figure 1:**
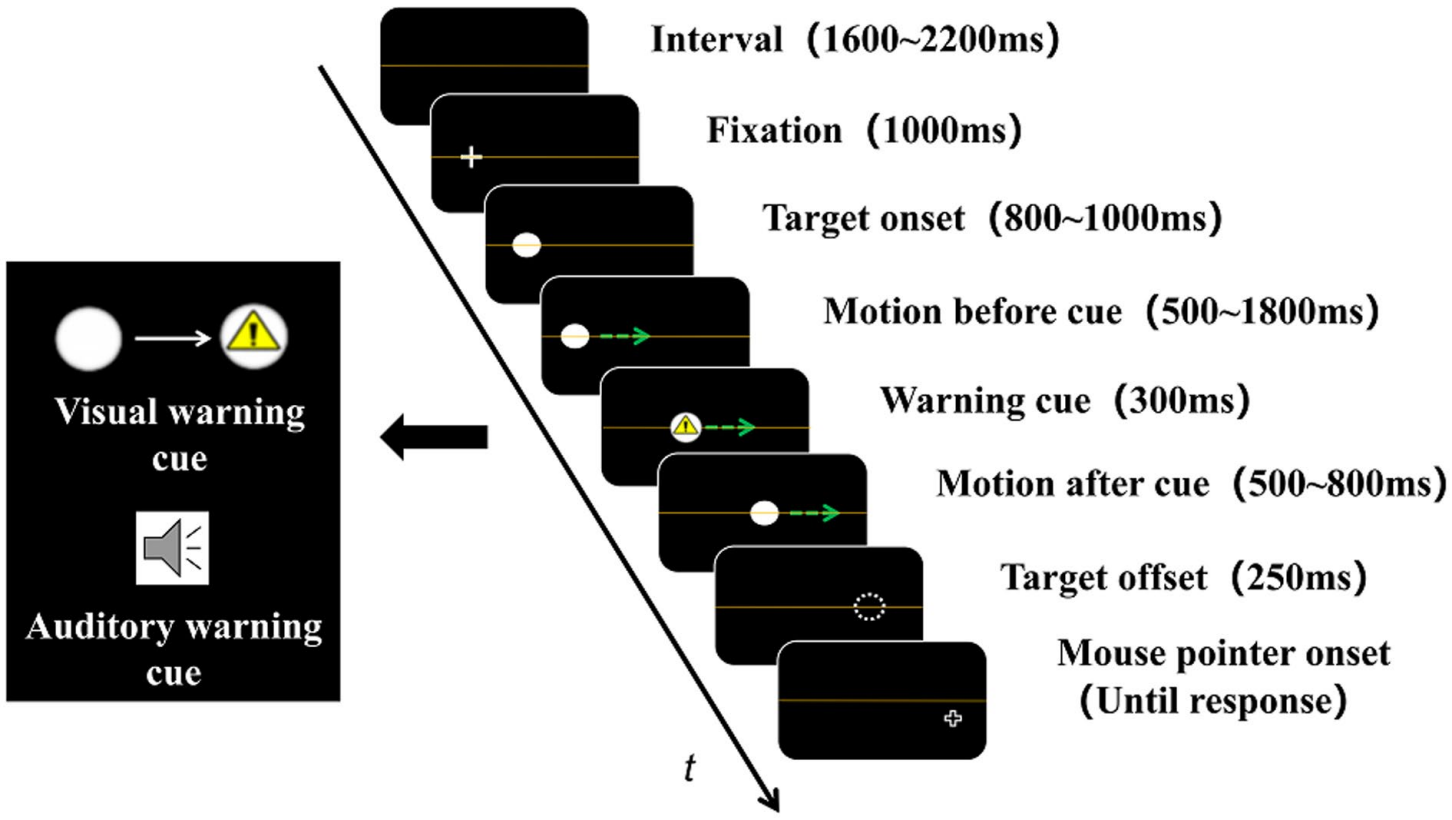
Experimental sequence. The green arrow in the picture indicates the direction of the target ball’s motion (absent during the experiment). The warning cue was presented during target motion. The visual cue was a triangle warning sign (above the black box on the left), whereas the auditory cue was a pure tone (below the black box on the left). In a cue-absent trial, neither the visual nor the auditory cue was presented.

The sequence of each trial is shown in Figure 1. There was an orange line indicating the height of the horizontal centerline, which was also the track through which the target ball moved. Each trial began with a 1,600–2,200 ms blank interval, and a cross fixation point appeared at an 11.32–17.34° random position on the left (when moved rightward) or right (when moved leftward) of the monitor and remained visible for 1,000 ms. Then, the fixation point vanished, and the target ball appeared at the same position and was static for 800–1,000 ms. After that, the target ball moved horizontally at a speed of 10.43°/s for 500–1,800 ms, and the subsequent warning cue was presented for 300 ms (if it was a cue-absent trial, then nothing happened). Then, the target ball vanished suddenly after continued movement for 500–800 ms. Next, a cruciform mouse pointer appeared after a 250-ms retention interval and remained visible until the observers moved it to the position where they thought the target ball actually vanished and pressed the left mouse button.

Before the beginning of the experiment, each observer was instructed to adjust their sitting posture and the position of the monitor to ensure that the sight line was level with the horizontal centerline of the monitor and asked to keep a distance of 50 cm from the monitor. The observers completed four cue-absent practice trials, and then they were told that visual or auditory warning cues would appear in the next trials to indicate the upcoming disappearance of the target ball. Then, the observer completed four cue-present practice trials and 12 comprehensive practice trials (the warning cues might or might not appear) to ensure that they fully understood the function of the cues. Afterward, they completed the formal experiment.

### Results

**Figure 2:**
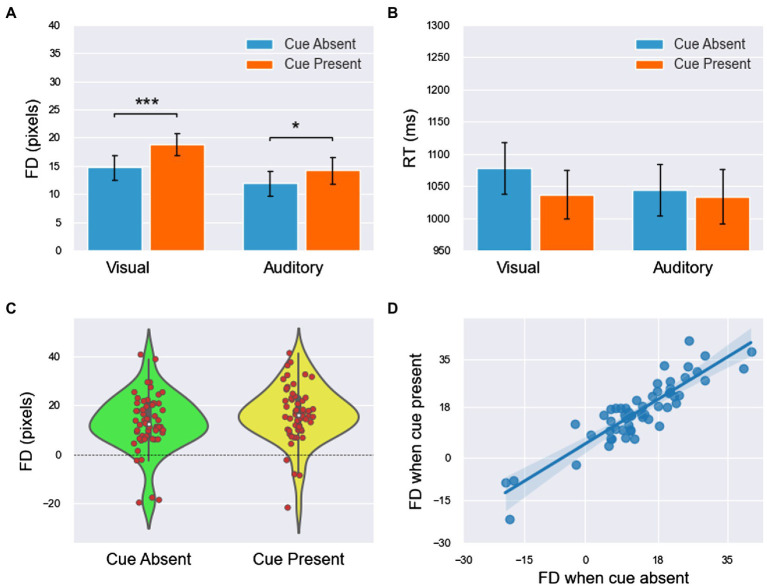
The FD and RT results of experiment 1 (all main effects are not labeled in the figure). **(A,B)** The means of FD and RT in the four conditions. **(C)** The FDs (including both visual and auditory blocks) were concentrated at higher values when the cue was present. **(D)** The linear relationship of FDs between the cue-absent and cue-present conditions had a slope of less than 1 and an intercept greater than 0. All significance level markings are as follows: ^*^p < 0.05, ^**^p < 0.01, ^***^p < 0.001.

For the purpose of the present study, localization biases in the direction orthogonal to the trajectory were not considered since they reflected biases to localize toward the fovea (Sheth and Shimojo, 2001; Kerzel, 2002) and the effects of the initial mouse pointer (van der Heijden et al., 1999; Kerzel, 2004). The dependent variables were FD, which was defined as the deviation along the direction of the target motion, and RT, which was the duration between when the mouse pointer appeared and when the response was executed. The trials in which FD or RT values exceeded three standard deviations were rejected; a total of 113 trials, 4.8% of the total, were rejected accordingly. The final FD and RT results are shown in Table 1 and Figure 2.

**Table 1:** The means and standard errors in all three experiments.

		**Visual Block**		**Auditory Block**	
		**No cue**	**Cue**	**No cue**	**Cue**
**Experiment 1** (*N* = 28)	**FD** (px)	14.42 ±2.19	18.91 ±1.93	11.88 ±2.23	14.18 ±2.31
	**RT** (ms)	1077.76 ±40.57	1036.77 ±38.22	1043.79 ±39.62	1033.54 ±42.67
					
**Experiment 2** (*N* = 31)	**FD** (px)	14.05 ±1.09	18.18 ±1.05	14.53 ±1.36	16.12 ±1.08
	**RT** (ms)	1208.93 ±60.41	1206.31 ±66.71	1218.37 ±63.43	1174.57 ±55.53
					
**Experiment 3** (*N* = 28)	**FD** (px)	14.93 ±2.17	20.81 ±1.63	13.92 ±2.20	15.74 ±2.05
	**RT** (ms)	564.77 ±20.73	550.37 ±17.99	562.24 ±21.10	541.13 ±20.47

For the combined analysis of the two blocks, 2 (modality: visual/auditory) × 2 (cue: absence/presence) ANOVA was used for both FD and RT, and the results showed the following:

1. For FD (Figure 2A), the main effect of modality was significant [ $F\left(1,27\right)=15.019,p<0.001,\eta_{p}^{2}=0.36$ ]. FD was significantly larger in the visual block than in the auditory block. The main effect of the cue was significant [ $F\left(1,27\right)=18.707,p<0.001,\eta_{p}^{2}=0.41$ ]. FD was significantly smaller in cue-absent trials than in cue-present trials (Figure 2C). The interaction between modality and the cue was insignificant [ $F\left(1,27\right)=3.499,p=0.072,\eta_{p}^{2}=0.12$ ].
2. For the RT (Figure 2B), the main effect of modality was insignificant [ $F\left(1,27\right)=0.617,p=0.439,\eta_{p}^{2}=0.02$ ]. The main effect of the cue was significant [ $F\left(1,27\right)=6.939,p=0.014,\eta_{p}^{2}=0.20$ ]. RT was larger in cue-absent trials than in cue-present trials. The interaction between modality and the cue was insignificant [ $F\left(1,27\right)=1.876,p=0.182,\eta_{p}^{2}=0.07$ ].

To further explore the difference in FDs between the cue-absent condition and the cue-present condition in the visual and auditory blocks, paired sample T tests were used in each block. The results showed that FD was significantly smaller in cue-absent trials than in cue-present trials in both visual block [*t*(27) = −4.435, *p* < .001, *Cohen’s d* = 0.41] and auditory block [*t*(27) = −2.438, *p* < 0.022, *Cohen’s d* = 0.19].

Then, the integrated data of the two blocks showed that FDs under the two conditions (cue-absent vs. cue-present) showed a high level of positive correlation ( ρ=0.931,p<.001, see Figure 2D). Moreover, the results of linear regression showed that there was a linear relationship between FDs with cue absent and present $\left[R_{a}^{2}=0.866,F\left(1,26\right)=168.505,p<0.001\right]$ . The slope was 0.892 $\left[t\left(26\right)=12.981,p<0.001,95\%\;CI=\left(0.750,1.033\right)\right]$ and the intercept was 4.824 [*t*(26) = 4.075, *p* <.001, 95% *CI* = (2.390,7.077)].

The results of one-sample T tests showed that the FD values under all four conditions were significantly far from zero [visual cue absent: $t\left(27\right)=6.570,p<0.001,\mathrm{Cohe}n^{\prime }s\;d=1.76$ ; visual cue present: $t\left(27\right)=9.799,p<0.001,\mathrm{Cohe}n^{\prime }s\;d=2.62$ ; auditory cue absent: $t\left(27\right)=5.336,p<0.001,\mathrm{Cohe}n^{\prime }s\;d=1.43$ ; auditory cue present: $t\left(27\right)=6.130,p<0.001,\mathrm{Cohe}n^{\prime }s\;d=1.64]\text{,}$ which suggested that representational momentum had occurred in all four conditions. The results of the independent sample T test indicated that the differences in FDs between male and female observers were insignificant [cue absent: $t\left(26\right)=-1.462,p=0.156,\mathrm{Cohe}n^{\prime }s\;d=0.63\text{;}$ cue present: *t*(26) = −1.216, *p* = .235, *Cohen’s d* = 0.55)].

### Discussion

Forward displacement was larger when both visual and auditory cues were present than when cues were absent, which indicated that phasic alertness boosts representational momentum rather than reduces it. This result is inconsistent with the results of Hubbard et al. (2009), but similar to the results of Kerzel (2003a). The cues that were used by Hubbard et al. and appeared during target motion either prompted observers’ alertness or hinted at the final location of the target, causing a decrease in FD. Combined with the present experimental results, it could be inferred that the decreases in FD occurred because the facilitation of alertness was smaller than the inhibition of the hint of the final location. This is easy to understand because alertness, a subcortical function (Peterson and Posner, 2012), is supposed to have a weaker effect than that of a location hint, which could directly strengthen observers’ memory of the final location since it is placed on that location during the entire period of movement. Therefore, the hint improved accuracy and decreased FD.

When the linear relationship of FDs between the cue-absent and cue-present conditions was shown, it was important to determine whether the slope was greater than 1. If so, that means alertness produced an add-effect on representational momentum. If it is less than 1, then the effect of alertness is limited by the observers’ inherent ability. In experiment 1, the slope was 0.892, but 1 was included in the 95% CI (0.750, 1.033), so the significance of this result is unclear. In addition, it appeared that the visual cue had a greater effect on FD than the auditory cue. Given that representational momentum is a visual location task, a cross-modality warning cue such as an audio cue might be less effective.

One might consider that the warning cue presented during target motion in experiment 1 might either produce alertness or disturb the action that observers were performing due to its sudden appearance. Past studies have found that observers’ RT to stimuli decreases under an alert state (Fan et al., 2002, 2005; Peterson and Posner, 2012), and if the cue disturbed the observers’ actions, RTs should increase. The results of experiment 1 showed a decrease in RTs with the cue present, which was consistent with the effect of alertness. Therefore, the warning cue used in experiment 1 resulted in alertness instead of a disturbance.

## Experiment 2

The evidence from experiment 1 proved that phasic alertness boosts representational momentum. It was found in past studies that when observers needed to attend to both target onset and offset, the effect of the speed of target motion on representational momentum decreased (Hubbard and Motes, 2002), which suggested a possible interaction between the effects of attention and speed on representational momentum. Although the authors did not statistically confirm this viewpoint, given that speed is the most stable of the factors that influence representational momentum (Hubbard, 2005), it was necessary to investigate whether attention would result in this variety. In experiment 2, the organization of warning cues was the same as in experiment 1, but the target ball moved at two different speeds to allow an investigation of the interaction between phasic alertness and speed.

### Methods

#### Participants

Thirty-four right-handed students from Southwest University of China were paid for their participation, but the data of three of these individuals were removed because the experimental system crashed during the experiment. Thus, data from 31 participants (27 females, mean age 20.61 ± 1.91 years) were included in the analysis. All had normal or corrected-to-normal vision. None reported any visual or auditory impairment. Before commencing the experiment, they signed the consent protocol, which was approved by the Ethics Committee of Southwest University. The result of the post-hoc power analysis showed 1−β>0.99 for the observed main effect of the cue with FD as dependent variable ( f=0.83,α=0.05,N=31, , run with G*Power 3; Luna et al., 2022a).

#### Stimuli and apparatus

These were the same as in experiment 1.

#### Design and procedure

The design and procedure were similar to those in experiment 1, but each block had two independent variables (warning cue: absent/present, speed: low/high). Each of these four conditions had 10 repetitions. The method of balancing moving directions and setting catch trials was the same as in experiment 1, so this experiment also had 84 trials in total. The target ball moved at a speed of 6.3°/s in the low-speed trials and at a speed of 11.64°/s in the high-speed trials.

### Results

**Figure 3:**
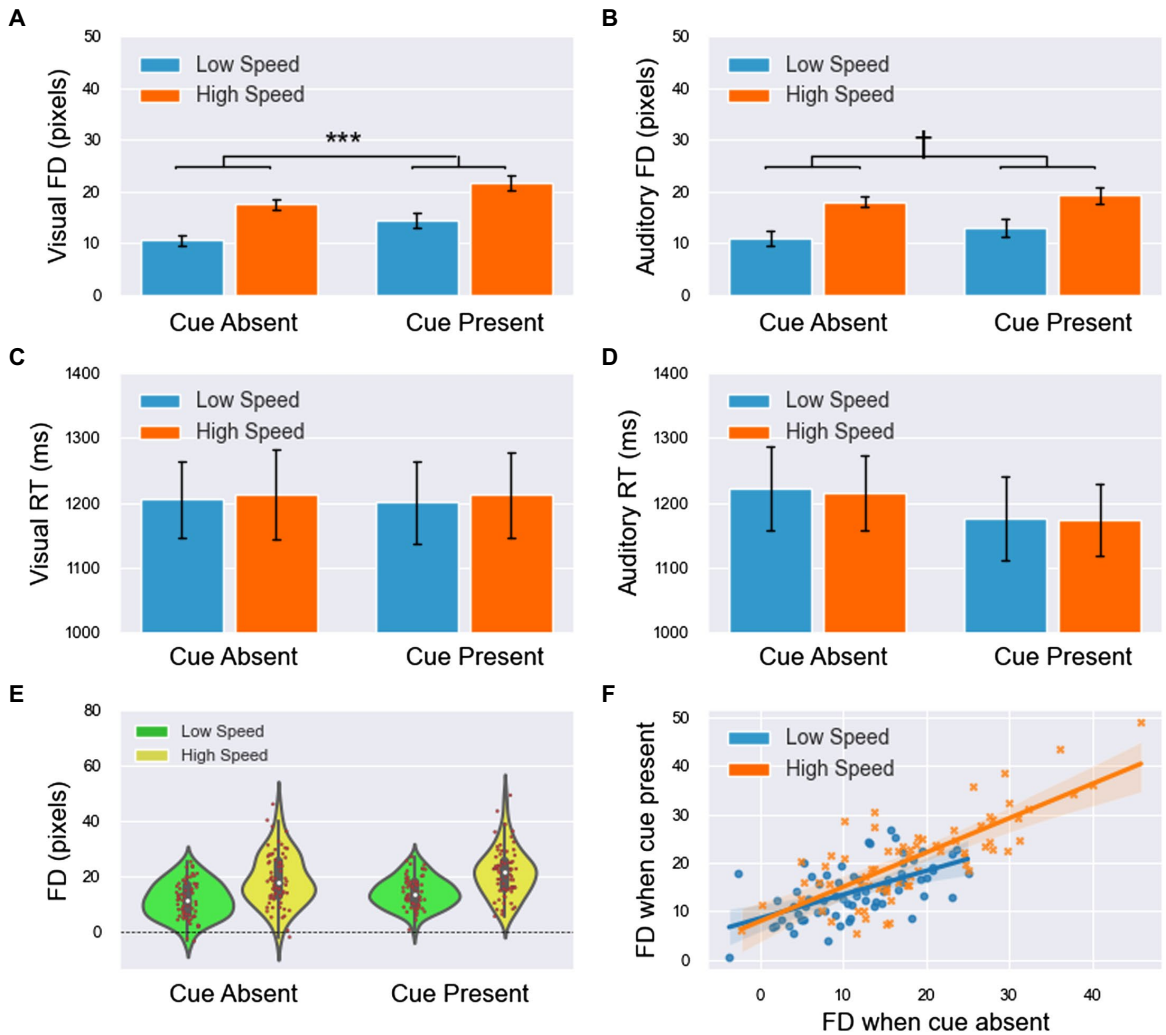
The results of forward displacement (FD) and response time (RT) in experiment 2 (all main effects are not labeled in the figure). **(A,B)** The means of FD in the visual and auditory blocks. **(C,D)** The means of RT in the visual and auditory blocks. **(E)** The cue shifted the distribution of FDs to higher values (including both visual and auditory blocks), and when the speed was high, the FDs were larger and more dispersed. **(F)** The linear relationship of FDs between the cue-absent and cue-present conditions had a slope of less than 1, which indicated that if observers’ mental extrapolation was easier when they were not alerted, then the effect of the alerting cue would be smaller. This finding suggested that the effect of the cue had an upper limitation. Moreover, the slopes of the two lines were roughly identical, which indicated that the effect of the cue on FD was not modulated by speed. All significance level markings are as follows: ^†^*p* < 0.1, ^*^p < 0.05, ^**^p < 0.01, ^***^p < 0.001.

A total of 133 trials, 5.4% of the total, were rejected by the same method used in experiment 1. The meanings of FD and RT were the same as in experiment 1, and the results are shown in Table 1 and Figure 3.

For the combined analysis of all blocks, 2 (modality: visual/auditory) × 2 (speed: low/high) × 2 (cue: absence/presence) ANOVA was used for both FD and RT, and the results showed the following information:

1. For the FD (Figures 3A,B), the main effect of modality was insignificant [ $F\left(1,30\right)=0.991,p=0.327,\eta_{p}^{2}=0.03$ ]. The main effect of speed was significant $\left[F\left(1,30\right)=50.146,p<0.001,\eta_{p}^{2}=0.63\right]\text{.}$ FD was significantly smaller in low-speed trials than in high-speed trials. The main effect of the cue was significant $\left[F\left(1,30\right)=20.610,p<0.001,\eta_{p}^{2}=0.41\right]\text{.}$ FD was significantly smaller in cue-absent trials than in cue-present trials (Figure 3E). The interaction between modality and the cue was significant $\left[F\left(1,30\right)=7.339,p=0.011,\eta_{p}^{2}=0.20\right]\text{.}$ The results of simple effect analysis showed that in the visual block, FD was significantly smaller when the cue was absent than when the cue was present ( p<0.001 ), whereas in the auditory block, this difference was not significant ( p=0.070 ).
2. For the RT (Figures 3C,D), the main effect of modality was insignificant [ $F\left(1,30\right)=0.183,p=0.672,\eta_{p}^{2}=0.01$ ]. The main effect of speed was insignificant [ $F\left(1,30\right)=0.044,p=0.835,\eta_{p}^{2}=0.001$ ]. The main effect of the cue was insignificant [ $F\left(1,30\right)=3.859,p=0.059,\eta_{p}^{2}=0.11$ ], but there was a tendency for RT to be larger in cue-absent trials than in cue-present trials (1,214 vs. 1,190 ms). All interactions were not significant ( p>0.05 ).

The integrated data of the two blocks showed that FDs under the two conditions (cue-absent vs. cue-present) were highly positively correlated ( ρ=0.811,p<0.001, see Figure 3F). The results of linear regression showed that there was a linear relationship between FDs in the absence and presence of the cue under both low-speed [ $R_{a}^{2}=0.510,F\left(1,29\right)=32.178,p<0.001$ ]and high-speed $\left[R_{a}^{2}=0.645,F\left(1,29\right)=55.622,p<0.001\right]$ conditions. When the speed was low, the slope was 0.591 [*t*(29) = 5.673, *p* <.001, 95% *CI* = (0.378,0.804)] and the intercept was 7.422 [*t*(29) = 5.813, *p* <.001, 95% *CI* = (4.810,10.033)]. When the speed was high, the slope was 0.776 [*t*(29) = 7.458, *p* <.001, 95% *CI* = (0.564,0.989)] and the intercept was 6.694 [*t*(29) = 3.317,*p* = 0.002, 95% *CI* = (2.567,10.821)].

### Discussion

The main effects of speed on FD were significant, thus indicating that FD increased with increasing speed, consistent with the results of past studies (Freyd and Finke, 1985; Freyd et al., 1990; Munger and Minchew, 2002; Munger and Owens, 2004). However, the interactions between warning cues and speed were not significant, which indicated that the effect of speed on representational momentum was not modulated by phasic alertness. This result further confirmed the stability of the effect of speed on representational momentum. Accordingly, regardless of whether low speeds or high speeds were used, FD increased in response to both visual and auditory cues, consistent with the results of experiment 1. This result suggested that the effect of phasic alertness on representational momentum is not modulated by speed. In addition, the RT results were consistent with those in experiment 1, which indicated that warning cues also produced an alert state.

In experiment 2, the slopes between cue-absent and cue-present FDs were less than 1 (1 was excluded from the 95% CI) under both low-speed and high-speed conditions, which verified the tendency observed in experiment 1. These results indicated that if observers’ mental extrapolation was easier when they were not alerted, then the effect of the alerting cue would be smaller, which suggested that the effect of the cue had an upper limitation. Moreover, the difference in the slopes between low speed and high speed was insignificant (the two 95% CIs overlapped), which further indicated that the effect of the cue on FD was not modulated by speed. However, high speed might also cause more attention, so this result should be interpreted with caution.

## Experiment 3

In general, when a smooth motion paradigm is used to investigate representational momentum, the mouse pointer paradigm is often used. This method was used in experiments 1 and 2 to prove that phasic alertness boosts representational momentum. However, the localization results might be affected by the error caused by the initial position of the mouse pointer. Moreover, previous studies on the effect of attention on representational momentum have all adopted the probe paradigm. Past studies have shown that differences in response methods can also influence representational momentum. Specifically, FD values are larger when a mouse pointer is used than when a probe is used (Kerzel, 2003b; Ashida, 2004). Therefore, whether the boost of representational momentum caused by phasic alertness still occurs when a probe is used needs to be investigated. In experiment 3, seven possible locations of a probe were used instead of the mouse pointer. FD was reflected by the point of subjective equality (PSE), which was calculated by cumulative Gaussian function fitting, and both the fitting standard deviation (SD) and fitting coefficient $R^{2}$ were acquired. FD, SD, RT, and accuracy (ACC) were analyzed in experiment 3.

### Methods

#### Participants

Thirty right-handed students (24 females, mean age 20.83 ± 1.62 years) from Southwest University of China were paid for their participation. Three of them were removed from analysis (see Results section) so 28 participants left. All had normal or corrected-to-normal vision. None reported any visual or auditory impairment. Before commencing the experiment, the participants signed the consent protocol that was approved by the Ethics Committee of Southwest University. The result of the post-hoc power analysis showed 1−β>0.99 for the observed main effect of the cue with FD as dependent variable ( f=0.88,α=0.05,N=28 , run with G*Power 3; Luna et al., 2022a).

#### Stimuli and apparatus

These were the same as in experiment 1.

#### Design and procedure

The design and procedure were similar to those in experiment 1, but there were seven possible probe locations: −40/−20/0/20/40/60/80 (pixels). Positive numbers indicated that the probe would appear on the side along the direction of target motion, whereas negative numbers signified that the probe would appear on the side opposite the direction of target motion. The absolute value represented the number of pixels between the probe and the target vanishing location. Each of these locations was repeated 12 times. As 12 catch trials were performed, experiment 3 had a total of 348 trials. The observer was asked to determine whether the location of the probe was on the left of, on the right of, or coincident with the location where the target vanished. If the observer determined that the location was to the left, then the observer pressed the button “←”; the observer pressed the button “→” was pressed if he or she determined that the location was to the right. If the observer determined that the location was coincident, then the button “↓” was pressed. The observers were asked to press the buttons as quickly as they could under the premise of ensuring correctness. Due to the additional number of trials, in addition to the 2-min rest period between the two blocks, the observer also took a 2-min rest period after the first half of trials in each block.

### Results

**Figure 4:**
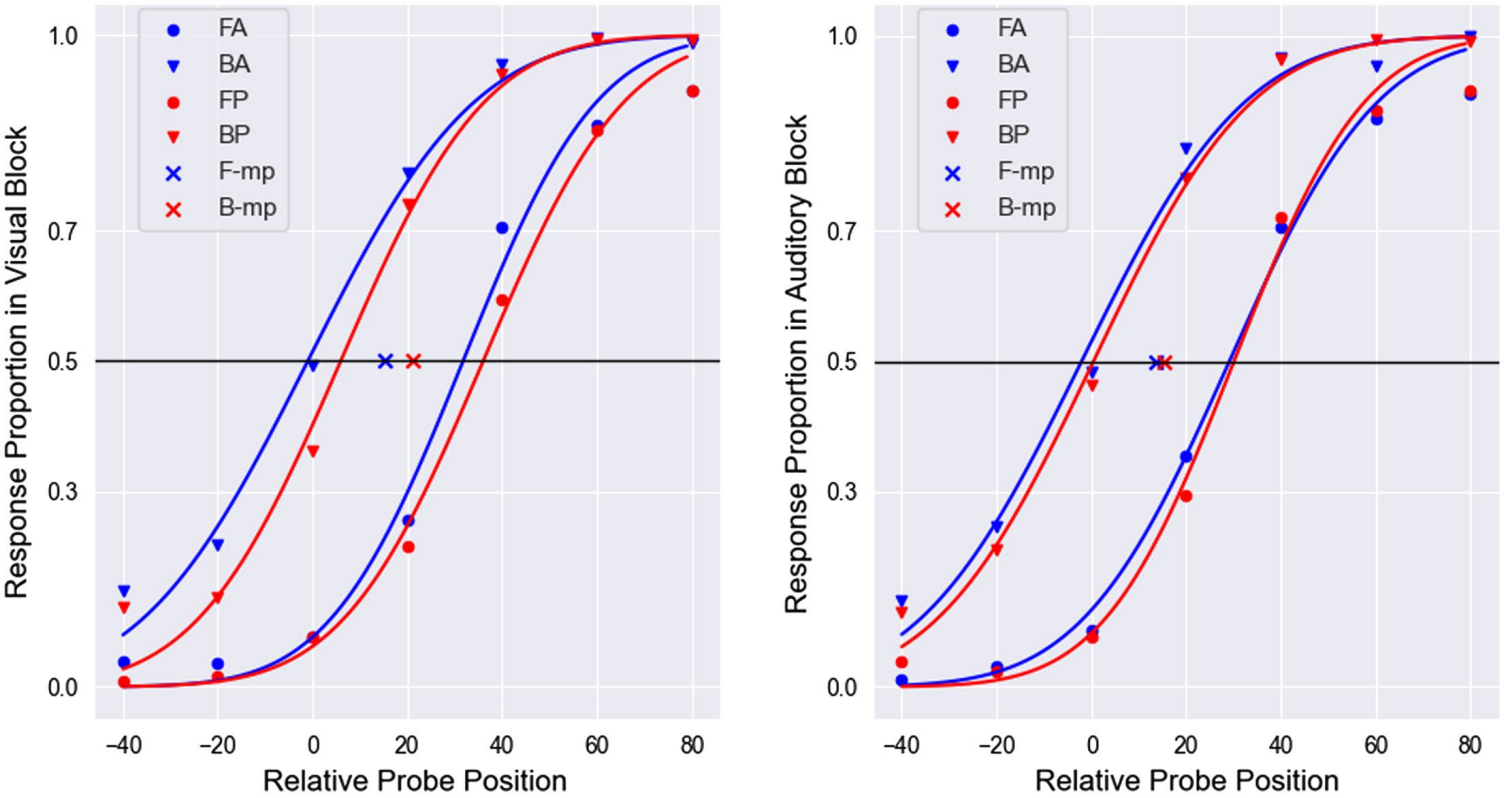
The proportion of responses at each probe position and their fitting curve in both the visual (left) and auditory (right) blocks. FA represents the forward proportion in the cue-absent condition. BA represents the 1-backward proportion in the cue-absent condition. FP represents the forward proportion in the cue-present condition. BP represents the 1-backward proportion in the cue-present condition. F-mp represents the midpoint between 0.5-height points on two red lines, and B-mp represents the midpoint between 0.5-height points on two blue lines. Taking “cue absent” as an example, the final PSE equals the abscissa of the blue “*x*.” It can be seen that in the visual block, the red “*x*” is shifted to the right compared with the blue “*x*,” which means the observers were less likely to perceive the probe in front of the target when the cue was presented, and overall, the red lines were steeper than the blue lines. These results indicated that the visual cue increased FD and decreased SD, which suggested that phasic alertness boosts representational momentum and increased attention.

**Figure 5:**
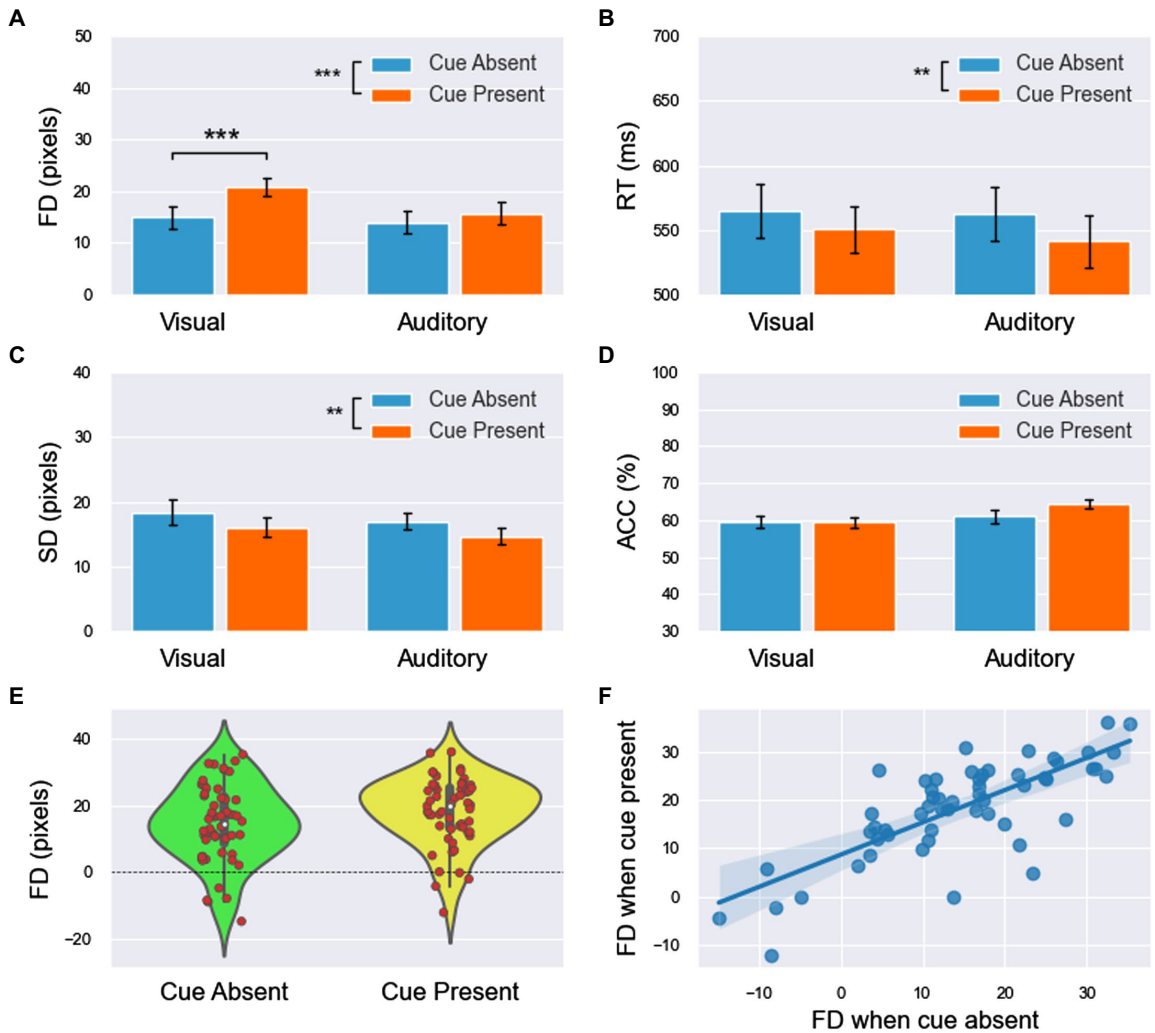
The results of forward displacement (FD, **A**), response time (RT, **B**), fitting standard deviation (SD, **C**), and accuracy (ACC, **D**) in experiment 3 (the main effect of modality is not labeled in the figure). **(E)** The cue shifted the distribution of FDs (including both visual and auditory block) to higher values. **(F)** The linear relationship of FDs between the cue-absent and cue-present conditions had a slope of less than 1. Similar to the results of experiment 2, it can be speculated that the effect of the cue had an upper limitation. All significance level markings are as follows: ^*^p < 0.05, ^**^p < 0.01, and ^***^p < 0.001.

A total of 146 trials (1.5% of the total) in which RT exceeded three SDs were rejected. Then, all trials in which the response was not “coincident” were marked in this way: if the observer determined that the probe was on the side along the direction of target motion, then the response was marked as “forward”; if the observer determined that the probe was on the side opposite the direction of target motion, then the response was marked as “backward.” The proportions of forward and backward in each condition were calculated, and the proportions of backward were further calculated to “1 minus proportion.” Four groups of data from each block were acquired: forward proportion with cue absent (FA), 1-backward proportion with cue absent (BA), forward proportion with cue present (FP), and 1-backward proportion with cue present (BP). Then, cumulative Gaussian function fitting was used on each proportion, which produced the fitting mean value that represented the PSE, also regarded as FD, fitting SD, and fitting coefficient $R^{2}$ . The final FD, SD, and $R^{2}$ with cues absent and cues present were acquired by calculating the mean forward and 1-backward values. SD represents the sensitivity to the difference between the target and probe positions. An increase in SD indicated a decrease in sensitivity. $R^{2}$ represents how well the data matched the normal pattern ranging from 0 to 1. In general, $R^{2}\geq 0.6$ was considered acceptable (Li et al., 2017, 2018). Two participants’ data were removed due to $R^{2}<0.6$ , which left 28 participants. The response proportions at each probe location and the fitting curves are shown in Figure 4, and the results of FD, RT, SD, and ACC are shown in Figure 5 (for FD and RT, see also Table 1).

For combined analysis of the two blocks, 2 (modality: visual/auditory) × 2 (cue: absence/presence) ANOVA was used for each measurement of FD, RT, SD and ACC, and the results showed the following information:

1. For FD (Figure 5A), the main effect of modality was significant [ $F\left(1,27\right)=4.278,p=0.048,\eta_{p}^{2}=0.14$ ]. FD was significantly larger in the visual block than in the auditory block. The main effect of the cue was significant [ $F\left(1,27\right)=20.990,p<0.001,\eta_{p}^{2}=0.437$ ]. FD was significantly smaller in cue-absent trials than in cue-present trials (Figure 5E). The interaction between modality and the cue was insignificant [ $F\left(1,27\right)=3.440,p=0.075,\eta_{p}^{2}=0.11$ ].
2. For RT (Figure 5B), the main effect of modality was insignificant [ $F\left(1,27\right)=0.249,p=0.622,\eta_{p}^{2}=0.01$ ]. The main effect of the cue was significant [ $F\left(1,27\right)=11.399,p=0.002,\eta_{p}^{2}=0.30$ ]. RT was significantly larger in cue-absent trials than in cue-present trials. The interaction between modality and the cue was insignificant [ $F\left(1,27\right)=0.875,p=0.358,\eta_{p}^{2}=0.03$ ].
3. For SD (Figure 5C), the main effect of modality was insignificant [ $F\left(1,27\right)=1.188,p=0.285,\eta_{p}^{2}=0.04$ ]. The main effect of the cue was significant [ $F\left(1,27\right)=8.190,p=0.008,\eta_{p}^{2}=0.23$ ]. SD was significantly larger in cue-absent trials than in cue-present trials. The interaction between modality and the cue was insignificant [ $F\left(1,27\right)=0.001,p=0.978,\eta_{p}^{2}=0.001$ ].
4. For ACC (Figure 5D), the main effect of modality was significant [ $F\left(1,27\right)=7.603,p=0.010,\eta_{p}^{2}=0.22$ ]. ACC was significantly lower in the visual block than in the auditory block. The main effect of the cue was insignificant [ $F\left(1,27\right)=1.994,p=0.169,\eta_{p}^{2}=0.07$ ]. The interaction between modality and the cue was insignificant [ $F\left(1,27\right)=3.650,p=0.067,\eta_{p}^{2}=0.12$ ].

A paired sample T test was used in both the visual and auditory blocks. The results showed that FD was significantly smaller in cue-absent trials than in cue-present trials in the visual block [ $t\left(27\right)=-4.724,p<0.001,\mathrm{Cohe}n^{\prime }s\;d=0.58$ ], whereas in the auditory block, this difference was not insignificant [ $t\left(27\right)=-1.213,p=0.236,\mathrm{Cohe}n^{\prime }s\;d=0.16$ ].

The integrated data of the two blocks showed that FDs under the two conditions (cue-absent vs. cue-present) were highly positively correlated ( ρ=0.902,p<0.001 , see Figure 5F). The results of linear regression showed that there was a linear relationship between FDs in the absence and presence of the cue [ $R_{a}^{2}=0.814,F\left(1,26\right)=114.096,p<0.001$ ]. The slope was 0.769 [ $t\left(26\right)=10.682,p<0.001,95\%CI=\left(0.621,0.917\right)$ ], and the intercept was 7.182 [*t*(26) = 5.666, *p* < 0.001, 95% *CI* = (4.577, 9.788)].

### Discussion

Using a probe paradigm, experiment 3 again proved that phasic alertness boosts representational momentum, thus providing replicable and consistent evidence using the current design. SD decreased when the cue was presented, which indicated that the observers’ sensitivity to the difference between the target and probe positions increased. This increased sensitivity would be an indication that the alert state was produced effectively.

In experiments 1 and 2, the RT decreased in the cue-present trials, but not all increases were significant, possibly because the observers were not strictly required to react rapidly when the mouse pointer paradigm was used. Past studies have shown that the decrease in RT caused by alertness is most obvious in tasks that require response speed (Peterson and Posner, 2012). In experiment 3, response speed was required, which led to a significant decrease in RT in both the visual and auditory blocks. However, these decreases might be caused by alertness, the speed-accuracy trade-off used by the observers, or both. If a speed-accuracy trade-off occurred, then a decrease in ACC should be observed, which was not observed in experiment 3. Therefore, the warning cue produced an alert state among the observers.

## General discussion

The present study investigated the effect of one of the three components of attention—phasic alertness—on representational momentum. In experiment 1, the mouse pointer paradigm was used and demonstrated that external warning cues increased FD. Experiment 2 proved that the effects of phasic alertness and speed of motion on representational momentum were independent. In experiment 3, the probe paradigm was used to further show that external warning cues increased FD and sensitivity to differences between the target and probe positions. The results of the present study were inconsistent with those reported by Hubbard et al. (2009), which suggested that in the spatial cue paradigm that Hubbard et al. used, cues might play dual roles of producing alertness and hinting at the final location of the target. The facilitation by the former was smaller than the inhibition by the latter, so FD decreased. In general, the results of the present study are similar to those of Kerzel (2003a).

How does alertness boost representational momentum? Presumably, this boost occurs because different components of attention are involved in different stages in the representational momentum task. During target motion, the observer needs to attend to the appearance of the probe (probe paradigm) or disappearance of the target (mouse pointer paradigm). The location of the former is uncertain, and both the location and time of the latter are uncertain. Thus, the observer must be prepared to respond to the event in advance. Therefore, regardless of whether the warning cue was present, the more commonly used component of attention during target motion was alertness, and the effect of the warning cue was simply to enhance alertness level in a short time window. This is easy to understand, as a sudden change in status of a moving object might cause danger; thus, human have learned to remain vigilant about any change in status of a moving object. In contrast, after the target suddenly vanished, due to the lack of a deceleration process, the sudden disappearance completely contradicted physical laws in the objective world; hence, it also completely contradicted the experiences of the observer, which caused a mental extrapolation. Researchers have found that FD exists even when an observer’s eye movements are limited; thus, FD can only be caused by the automatic mental extrapolation process (Kerzel, 2003a). However, this automatic process conflicts with the desired action—remembering the true location where the target disappeared. The observer needs to focus on overcoming this automatic process to complete the action. Therefore, the more commonly used component of attention during the retention interval is executive control.

In the representational momentum task, FD depended on the final judgment or localization, which is highly relevant to the capability to overcome automatic mental extrapolation in the retention interval. FD values are smaller when this executive control process is easier to execute and larger when it is more difficult. Many studies have shown that alertness can inhibit executive control performance by highlighting priority information and enhancing the automatic process (Van Vleet et al., 2011; Weinbach and Henik, 2011, 2012, 2013; Weinbach et al., 2015). The warning cue presented during the target motion enhanced the alert state, and thus enhanced the automatic mental extrapolation process after the target disappeared, further leading to an increase in FD and boosting representational momentum. This outcome might resolve the debate on whether attention is used to halt or maintain mental extrapolation raised by Hayes and Freyd (2002) and Kerzel (2003a) respectively. More specifically, the attention component used during target motion—alertness—is used to maintain mental extrapolation, and the attention component used during retention interval—executive control—is used to halt mental extrapolation. The dual tasks that Hayes and Freyd used might have influenced the executive control process, so the FD values increased. The distractors that Kerzel (2003a) and Hubbard et al. (2009) presented in the retention interval might have influenced executive control by completely transferring attention to themselves due to their sudden appearance; attention then returned to the original action. This process of attention transfer might have directly eliminated the alert state arising from the stage of target motion. As a result, the next executive control process was less affected by alertness, which allowed the observer to overcome automatic mental extrapolation more easily and ultimately decreased FD.

In addition, experiments 2 and 3 found that the effect of the cue on representational momentum had an upper limitation. Specifically, if the observer produced a large FD when they were not alerted, then the alerting cue had little influence on them. This might be because of their high inherent vigilance, which made it difficult to improve their alert state by using an external warning cue.

The present study suggests that different attention components are used in different stages in representational momentum. Few studies have focused on the temporal course of nerve action in representational momentum. Future studies could investigate the effects of attention in different stages by examining different nerve actions during the target motion and retention intervals.

## Conclusion

Phasic alertness boosts representational momentum, possibly by facilitating automatic mental extrapolation. The effect of phasic alertness is not modulated by speed.

## Data availability statement

The raw data supporting the conclusions of this article will be made available by the authors, without undue reservation.

## Ethics statement

The studies involving human participants were reviewed and approved by the ethics committee of Southwest University. The patients/participants provided their written informed consent to participate in this study.

## Author contributions

PL, JY, and JZ contributed to conception and design of the study. JY organized the database, performed the statistical analysis, and wrote the first draft of the manuscript. PL and JZ wrote sections of the manuscript. All authors contributed to the article and approved the submitted version.

## Funding

This research was supported by the National Natural Science Foundation of China (31600879), General Financial Grant from the China Postdoctoral Science Foundation (2015M582488), the Fundamental Research Funds for the Central Universities (SWU1509450; SWU1509451), the Grant from the Mechanism and Application of Temporal Range/Synthetic Model (TR201201-1), the Base Project of Humanities and Social Sciences Research of Chongqing (16SKB009), and the Special Grant of Postdoctoral Research Project of Chongqing (Xm2016088).

## Conflict of interest

The authors declare that the research was conducted in the absence of any commercial or financial relationships that could be construed as a potential conflict of interest.

## Publisher’s note

All claims expressed in this article are solely those of the authors and do not necessarily represent those of their affiliated organizations, or those of the publisher, the editors and the reviewers. Any product that may be evaluated in this article, or claim that may be made by its manufacturer, is not guaranteed or endorsed by the publisher.
